# CONTRAIS: CONservative TReatment for Adolescent Idiopathic Scoliosis: a randomised controlled trial protocol

**DOI:** 10.1186/1471-2474-14-261

**Published:** 2013-09-05

**Authors:** Allan Abbott, Hans Möller, Paul Gerdhem

**Affiliations:** 1 Department of Clinical Science, Intervention and Technology, Karolinska Institutet, Stockholm, Sweden; 2 Department of Physiotherapy, Karolinska University Hospital, Stockholm, Sweden; 3 Department of Orthopaedics, Karolinska University Hospital, Stockholm, Sweden; 4 Faculty of Health Science and Medicine, Bond University, Gold Coast, Australia

**Keywords:** Idiopathic scoliosis, Treatment, Brace, Exercise

## Abstract

**Background:**

Idiopathic scoliosis is a three-dimensional structural deformity of the spine that occurs in children and adolescents. Recent reviews on bracing and exercise treatment have provided some evidence for effect of these interventions. The purpose of this study is to improve the evidence base regarding the effectiveness of conservative treatments for preventing curve progression in idiopathic scoliosis.

**Methods/design:**

Patients: Previously untreated girls and boys with idiopathic scoliosis, 9 to 17 years of age with at least one year of remaining growth and a curve Cobb angle of 25–40 degrees will be included. A total of 135 participants will be randomly allocated in groups of 45 patients each to receive one of the three interventions.

Interventions: All three groups will receive a physical activity prescription according to the World Health Organisation recommendations. One group will additionally wear a hyper-corrective night-time brace. One group will additionally perform postural scoliosis-specific exercises.

Outcome: Participation in the study will last until the curve has progressed, or until cessation of skeletal growth. Outcome variables will be measured every 6 months. The primary outcome variable, failure of treatment, is defined as progression of the Cobb angle more than 6 degrees, compared to the primary x-ray, seen on two consecutive spinal standing x-rays taken with 6 months interval. Secondary outcome measures include the SRS-22r and EQ5D-Y quality of life questionnaires, the International Physical Activity Questionnaire (IPAQ) short form, and Cobb angle at end of the study.

**Discussion:**

This trial will evaluate which of the tested conservative treatment approaches that is the most effective for patients with adolescent idiopathic scoliosis.

**Trial registration:**

NCT01761305

## Background

Adolescent idiopathic scoliosis (AIS) is a three-dimensional structural deformation of the spine and trunk with lateral shift and rotation of the vertebrae [1]. Idiopathic scoliosis with curvature over 10° affects around 3% of children and adolescents. Idiopathic scoliosis occurs in otherwise healthy children and is often diagnosed at the time of the pubertal growth spurt. About one tenth develop a more aggressive variant leading to a more severe deformity of the spine and thorax [2]. By early adulthood, the majority of patients with scoliosis suffer from back pain and if the curve progresses to be very large, even pulmonary dysfunction and psychological distress can occur [3].

In its mild form, idiopathic scoliosis is common and is not treated. In Sweden, screening occurs in school grade 4 (age 10–11) and in grade 7 or 8 (ages 13–14), and it can be estimated that at least 200,000 children are screened yearly by school nurses and physicians. The aim is to find the children with moderate scoliosis. These are often treated with a brace to prevent progression to severe scoliosis. More severe curves are treated with spinal fusion surgery. Surgical complications are few but if they occur, may be devastating, such as paraplegia [4]. The spinal curve is corrected and fused with limitation in mobility as a result.

Several theories propose that during the adolescent period of skeletal growth, bone deformation may occur in the event of vertebral body weakness or an imbalance of muscle forces and joint flexibility [5]. A recent review of literature concerning the association of low bone mineral density and idiopathic scoliosis reported generalized osteopenia and an osteoporosis prevalence of 20–38 percent [6]. Adequate levels of self-mediated physical activity and intake of calcium and vitamin-D is a requirement for normal skeletal growth and development during childhood and adolescence. It is well documented that physical exercise is associated with improvements in not only muscle strength, aerobic fitness and motor development but also bone density which may help decrease the risk of osteopenic related bone deformation [7,8].

Current guidelines from the International Scientific Society on Scoliosis Orthopaedic and Rehabilitation Treatment (SOSORT) recommend physical therapy from curve magnitudes >15° Cobb [9]. Furthermore it is recommended that physical therapy and/or bracing of conservative treatment for AIS be implemented when curve magnitudes of 25-45° are apparent [9]. Surgical intervention for AIS is generally first considered when curve magnitudes reach >50° [10]. A recent systematic literature review of all available literature investigating the effectiveness of physical exercise in the treatment of AIS reported the results of 1 randomised controlled trial, 9 prospective cohort controlled trials, 8 prospective observational cohort studies and 2 retrospective cohort studies [11]. Together, the studies provide an evidence base of limited scientific quality suggesting the efficacy of physical exercise in reducing the scoliosis progression. Improved scientific quality of future research will better define the evidence base effects of physical therapies [12].

A recent literature review investigating the effectiveness of brace treatment of AIS reported the results of two prospective cohort controlled trials and 18 longitudinal case control studies [13]. Together, the existing studies provide an evidence base of limited scientific quality for the efficacy of bracing preventing the progression of scoliosis. Two randomised trials comparing brace treatment with observation only are currently being conducted; NCT00448448 and NCT01370057, registered at http://www.clinicaltrials.gov (accessed at March, 12th, 2013). Another trial from Holland has been terminated due to recruitment difficulties [14].

The on-going randomised trial use a thoracolumbar sacral orthosis worn during 20–23 hours per day as the active treatment. This brace is made of hard plastic and stretches from under the arms to the pelvis. It is custom made and corrects the scoliotic curvature of the spine when worn. The psychological impact when using the brace 20–23 hours per day should not be underestimated. In one study, 27% of the brace treated patients reported that the treatment had a major negative effect on their lives [15], and our clinical impression is that this is one of the reasons to the poor compliance that is often seen. Recently, preliminary data suggested that approximately eight hours of night-time bracing with an over-corrective brace was as effective as bracing during 23 hours per day [16].

Night-time bracing is attractive since you wear the brace a limited amount of time. The brace does not restrict activities during daytime. Our clinical impression is that the psychological concern for a teenager is much less when compared to brace treatment day and night-time, which increases the possibility of good compliance. There have been no controlled studies on night time bracing versus observation only. Several uncontrolled trials have been published, indicating an immediate corrective effect on the scoliosis by the brace [17].

Only one low quality study has compared bracing with physical exercise showing no statistical differences in the reduction or progression of scoliosis curves between the groups [18]. To draw valid conclusions about the effectiveness of conservative treatments for AIS, a randomised controlled trial research design is needed to compare the interventions.

### Aim of study

The primary objective of this study is to improve the evidence base regarding the effectiveness of conservative treatments for preventing the progression of AIS. Secondary aims include improving knowledge of clinical features that may predict a patient's response to each treatment.

### Hypotheses

1. That the use of a night-time brace and adequate levels of self-mediated physical activity is more effective in preventing curve progression than adequate levels of self-mediated physical activity alone.

2. That prescribing scoliosis specific exercise and adequate levels of self-mediated physical activity is more effective in preventing curve progression than adequate levels of self-mediated physical activity alone.

## Methods/design

### Study design

**Figure 1:**
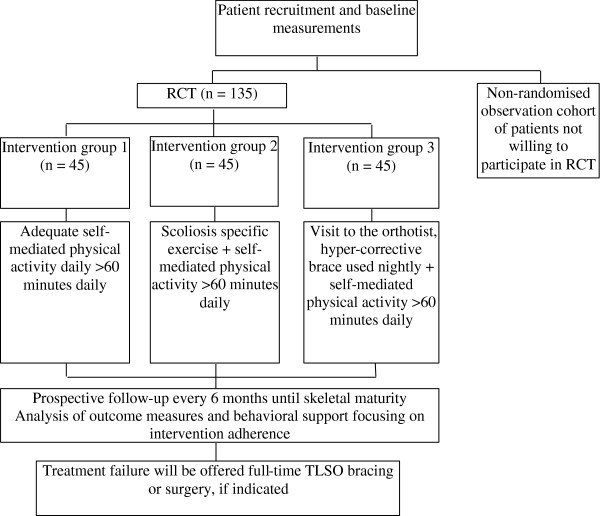
Flow chart of the randomised controlled study.

Prospective randomised controlled trial. In total, 135 patients will be randomised into one of three groups. The study design is outlined in Figure 1. The protocol conforms to the CONSORT guidelines for non-pharmacological studies [19] and has been registered on ClinicalTrials.gov, identifier: NCT01761305.

### Study centres

This is currently a single-centre study at Karolinska University Hospital in Stockholm, Sweden. Further study centres may be involved if the recruitment rate is slow. Children and adolescents that are referred to the orthopaedic department for scoliosis will be screened. Individuals that fulfil the inclusion criteria will be asked for participation. After informed consent, randomisation will take place.

### Inclusion criteria

– Diagnosis of idiopathic scoliosis.

– Skeletally immature with estimated remaining growth for at least one year.

– Not more than one year after menarche.

– Primary Cobb angle between 25 and 40 degrees.

– Curve apex T7 or caudal.

– Males and females aged 9–17 years.

### Exclusion criteria

– Scoliosis with a possible non-idiopathic aetiology. Patients will be excluded from the study if the pathogenesis of the scoliosis is not idiopathic, but due to a neuromuscular, neurological, congenital malformation or trauma related comorbidity.

– Previous brace or surgical treatment for scoliosis.

– Inability to understand Swedish.

### Randomisation

Randomisation will be done in a 1:1:1 ratio. A computer generated random numbers table in varying block sizes will be prepared a priori by an independent statistician. To conceal randomisation, the same independent statistician will prepare consecutively numbered, sealed opaque envelopes. The envelopes will be kept in a locked location. The envelope is to be opened in the presence of at least two persons. The original randomisation list will be kept by the independent statistician. One additional copy of the randomisation list is kept in a locked safe at the Karolinska Institutet, and not accessible for the research personnel.

### Blinding

In this study, blinding of patients and therapists for treatment is not possible. However, the randomization status of the participants will not be disclosed to the two physicians who will assess the patient's X-rays after the end of the study.

### Radiology

Standing standard scoliosis images will be performed. If possible, the same equipment will be used at all follow-ups. Anteroposterior or posteroanterior images will be performed depending on the laboratory equipment. The patient is asked to stand comfortably in a relaxed position. For the lateral image the patient is asked to stand comfortably in a relaxed position. The feet are parallel to the screen and beside each other. The right part of the body faces the radiation source. The patient holds his hands on a bar in a position in front of and above shoulder level, so that the arms do not obscure the vertebral column. Shoulder and elbow should be at the same level. The elbows are in a right angle. The hands are to be placed adjacent to one another. The brace is not to be worn during the night before the scoliosis images are taken. In addition, skeletal age will be determined from a conventional x-ray of the hand.

### Intervention groups

#### ***Group 1: adequate self-mediated physical activity***

Instructions for self-mediated physical activity will be delivered during an individual 1 hour session. Patients are encouraged to perform the self-mediated physical activities of moderate intensity at least 60 minutes daily, for the entirety of the study. Reinforcement of the assigned intervention will be performed in conjunction with reassessment every 6 months. A training diary will be implemented to follow and motivate the patient's compliance to the intervention protocol.

#### ***Group 2: scoliosis specific exercises***

In addition to receiving the same intervention as group 1, patients will learn how to perform a 3 dimensional self-mediated correction of their scoliosis, muscular stabilization of the corrected posture, and how to perform these postural correction strategies in their activities of daily living [20]. Additional self-mediated hyper-corrective exercises are to be performed with a dosage of 10 repetitions of 30 second isometric holds daily. The scoliosis specific exercises cover similar concepts and methods described in previous literature [21]. The intervention will be delivered to the patient individually by experienced physiotherapists in 3 × 90 minute sessions, once per month during the first 3 months and then every 6 months thereafter. Additional single bolus sessions may occur when extra education is required to master the program. Reinforcement of the intervention will be performed in conjunction with reassessment every 6 months. A training diary will be implemented to follow and motivate the patient's compliance to the intervention protocol.

#### ***Group 3: hyper-corrective night-time brace***

In addition to receiving the same intervention as group 1, a hyper-corrective brace will be worn 8 hours per night. The brace will be specifically designed to provide a 3 dimensional hyper-correction of the patient’s individual scoliosis type. Reinforcement of the assigned intervention will be performed in conjunction with reassessment every 6 months. A training diary will be implemented to follow and motivate the patient's compliance to the intervention protocol. The spine orthotist is available for brace adjustment when needed.

Patients in all groups are also be recommended 4 standard servings of dairy (or equivalent non-dairy substitute) daily to guarantee recommended daily intake of calcium and vitamin-D.

An additional file shows the content of the interventions this in more detail [see Additional file 1].

### Non-participants

Patients not willing to participate in the study will be offered the standard care of treatment, which in our institution is a corrective thoraco-lumbar sacral orthosos (TLSO) bracing for at least 20 hours per day. Patients not willing to participate in the study will be asked to fill in the same questionnaires as the study cohort at the time when treatment is suggested. The radiological and clinical result of these patients will be obtained from the regular orthopaedic files and radiographs taken.

### End of study

The study will be terminated when the participant reaches skeletal maturity, defined as less than 1.0 cm growth of body height in 6 months, or if the curve progresses more than 6 degrees, compared to the primary x-ray, seen on two consecutive spinal standing x-rays taken with 6 months interval. Patients who have a progression of the Cobb angle of more than 6 degrees and ultimately reaching the end point failure of treatment will not remain in the study but will be offered a TLSO brace treatment if indicated. In the event that a Cobb angle surpasses 50 degrees, patients will be offered surgical treatment. Post-treatment follow-up is planned to occur at 2, 5 and 10 years prospectively.

### Ethical permit

The trial has received ethical approval from the Regional Ethics Committee in Stockholm, Sweden (Dnr:2012/172-31/4).

### Funding

Funding for this study has been obtained from The Swedish Research Council (Dnr 521-2012-1771) and the regional agreement on medical training and clinical research (ALF) between Stockholm County Council and Karolinska Institutet (Dnr LS 1112–1587).

### Outcome measures

The patients will be followed up every 6 months for the entirety of the study.

#### ***Primary outcome measures***

• The primary outcome variable, failure of treatment, is defined as progression of the Cobb angle more than 6 degrees, compared to the primary x-ray, seen on two consecutive spinal standing x-rays taken with 6 months interval [22].

#### ***Secondary outcome measures***

• SRS-22r quality of life questionnaire [23].

• International Physical Activity Questionnaire (IPAQ) short form [24].

• EQ5D /-Y/ quality of life questionnaire [25].

• A modified Spinal Appearance Questionnaire [26].

• Cobb angle at end of the study.

At each 6 month follow-up additional questions regarding protocol fulfillment (According to CONSORT guidelines) [19], patient satisfaction and adverse effects will be asked. Body height and weight will be measured at baseline and at all follow-ups.

### Sample size

Based on figures from previous literature, a failure rate of 45% in the self-mediated physical activity group and a 15% failure rate in the brace and scoliosis specific exercise groups is hypothesised. Given a significance level of 5%, a power of 80% and consideration for dropout of up to 20%, an estimated 45 patients are required in each of the intervention groups.

### Data integrity

The integrity of trial data will be ensured by regularly monitoring of data sheets for omission and errors. Data will be double entered and the cause of any inconsistencies will be rectified. A minimum of 50% of the intervention quota is required to be fulfilled to be considered a compliant intervention.

### Data analysis

Data from the different groups will be compared based on the 'intention to treat' principle. An intention to treat (ITT) analysis means that all patients, regardless of noncompliance, loss to follow-up or drop-out, remain in the analysis of the group to which they were randomised [27]. Multiple imputation of missing data will be used in the ITT analysis. A sensitivity analysis will be performed comparing the ITT data against a per-protocol data exclusively from patients who complied with the study protocol. If a patient in the study receives full-time bracing or surgery due to treatment failure, the Cobb angle at the moment of commencing this treatment will be considered in the secondary outcome analysis of Cobb angle.

Categorical parameters will be compared by the Chi-square test. Continuous and discrete parameters will be measured using parametric or non-parametric tests (depending on skewness) for group comparisons. Individual variables will be tested for their association with treatment effect by adding a predictor × treatment group interaction term to a regression equation. Additionally, Kaplan-Meier survival analyses will be used to display the probability of more than 6 degree Cobb progression over time for each group.

## Discussion

The trial includes key methodological features that have been recognised as minimising bias in clinical trials: true randomisation, concealed allocation, specification of eligibility criteria, blind outcome assessment, blind analysis and intention-to-treat analysis. The nature of the treatments however precludes blinding of patient and treatment provided. Another potential source of bias is that there is no comparison of treatment groups to a randomised non-treatment control group to enable analysis of true effect of the treatments. This is because it can be considered unethical not to offer an active treatment to the eligible study population.

Our choice of outcomes is consistent with best practice recommendations for clinical trials studying AIS. The generalisability of future results may however be affected by our choice of bracing method. Our reasoning for using 3 dimensional hyper-corrective night–time bracing rather than full time bracing is that it may have potentially equivalent clinical effectiveness and possibly enable better compliance. It is also our clinical impression that the psychological concern for a teenager is much less when using a night-time brace compared to full-time use.

Another potential issue regarding generalisability is the content of the scoliosis specific exercise intervention. Some clinicians may advocate their preference for specific techniques based on their clinical experience or the limited scientific quality of previous studies. We have however taken an impartial approach to synthesizing a broad intervention covering principles outlined in scoliosis specific exercise literature. Future results from our scoliosis specific exercise protocol can only be generalised to the broad delivery of scoliosis specific exercise in an outpatient setting rather than generalised to individual techniques or application in an inpatient setting. Additionally, the study’s future results cannot be interpreted to suggest the potential efficacy of interventions involving both bracing and scoliosis specific exercise.

### Summary

This study uses a randomised controlled design to investigate the effectiveness of conservative treatments for preventing progression of AIS and the need for surgical interventions. The study also will investigate clinical features that may predict a patient's response to each treatment. The novel findings will enable evidence-based recommendations as to the effect of conservative interventions for AIS. Furthermore, findings will provide direction for future research into pathophysiological treatment rationale for the interventions.

## Competing interests

The authors have no competing interests to declare.

## Authors’ contributions

AA, PG conceived the project. PG, AA is leading the co-ordination of the trial. PG, AA, HM procured project funding. AA, PG, HM assisted with the protocol design. All authors provided feedback on drafts of this manuscript and have read and approved the final paper. All authors read and approved the final manuscript.

## Pre-publication history

The pre-publication history for this paper can be accessed here:

http://www.biomedcentral.com/1471-2474/14/261/prepub

## Supplementary Material

Additional file 1 Detailed description of interventions. Click here for file
