# Supplementary material for: CONTRAIS: CONservative TReatment for Adolescent Idiopathic Scoliosis: a randomised controlled trial protocol
Source: BMC Musculoskelet Disord. 2013 Sep 5;14:261. doi: 10.1186/1471-2474-14-261 (PMC3844437; doi:10.1186/1471-2474-14-261)
Supplement: Additional file 1 — Detailed description of interventions. [file 1471-2474-14-261-S1.docx]

**Detailed description of interventions**

The core components facilitating the delivery of each intervention is a cognitive-behavioural approach encompassing individual behavioural contracting, goal setting, self-monitoring, and reinforcement. Each individual participant with the help of the therapist will formulate their own behavioral strategies for implementing and adhering to the intervention protocol in their daily life. The therapist will help define the goal intervention dosages, and how they can be attained in a realistic and timely fashion. The therapist facilitated patient training sessions will utilise both repetitive implicit procedural learning strategies and explicit experiential learning of the intervention protocol through awareness, attention and reflection. This will allow the patient to acquire the skills to follow the intervention protocol independent of the therapist in their own daily living. Self-monitoring of progression through maintaining a diary account of their adherence to the intervention and the eventual attainment of each self-determined goals will provide intrinsic motivation for the patient. Prospective follow-up visits to the therapist every 6 months and contact via telephone when needed will provide a forum for extrinsic reinforcement of behaviors facilitating goal attainment. This can help to motivate the patient according to operant conditioning theory. Motivation strategies to optimise self-efficacy, which is one’s belief in completing a task, and positive outcome expectations, are essential to facilitate and maintain a behavioural change. Therapist’s feedback in the form of giving praise for successful progression and completion of tasks will provide a source of social persuasive and mastery experience for the patient, thus improving their self-efficacy and outcome expectation. If patients struggle to follow the requirements of the intervention program and to meet the goal dosage, new behavioral strategies will be formulated. Adjusting the individual prescription of the intervention and creating new goals to enhance and maintain motivation will occur when needed either via telephone contact with the therapist or additional booster outpatient therapy sessions.

*1. Adequate self-mediated physical activity*

Instructions for self-mediated physical activity will be delivered during an individual 1 hour session. Patients are encouraged to perform the self-mediated physical activities of moderate intensity at least 60 minutes daily, for the entirety of the study. Reinforcement of the assigned intervention will be performed in conjunction with reassessment every 6 months. A training diary will be implemented to follow and motivate the patient's compliance to the intervention protocol.

*2. Scoliosis specific exercise*

In addition to receiving the same intervention as group 1, patients in the scoliosis specific exercise group will receive additional intervention focusing on patient education of active self-correction in 3 dimensional planes (3D), muscular stabilization of the corrected posture, and application to activities of daily living. The intervention will be delivered individually in 3 x 90 minute sessions, once per month during the first 3 months and then every 6 months thereafter. Additional single bolus sessions may occur when extra education is required to master the program.

Motor learning can be described as a process associated with practice or experience leading to a behavioural change in the capability to produce a skilled action. The goals at the biomechanical and neuromotor levels are directed towards the patient’s learning of optimal correction of scoliosis curvature in 3D through muscle activation and postural movement strategies (self-correction). Furthermore the goals at the bodily function and psychological levels are directed towards a behavioural implementation of self-correction strategies in activities of daily living (ADL’s) and the development of a positive body image. The core content of the intervention sessions are as follows:

Session 1

- Education of body posture and awareness of postural deficits on the 3 spatial planes by using visual (mirror) and tactile (contact in the various postures) and verbal (therapist) feedback.
- Learning active self-correction on the 3 spatial planes:
  - Training awareness of correction on the sagittal plane to ensure thoracic kyphosis and lumbar lordosis within normal ranges. The patients train pelvic anteversion and a kyphotisation movement at the thoracic level by ventralising the lower ribs. The patient is given feedback from the therapist with the amount of corrective movement required in the sagittal plane to produce an apex of the lumbar lordosis at L2. This is trained in sitting and standing initially with tactile support and verbal cues for feedback. When the patient becomes more aware of the movement, it is then done with less feedback.
  - Training of the awareness of curve apex translation towards concavity on the frontal plane to attain more symmetrical vertebral column alignment. For example, in the case of a single-curve scoliosis, teaching how to execute thoracic curve horizontal translation through shoulder girdle retraction or in the case of a lumbar curve horizontal side shift in relation to the pelvis. In the case of a thoracolumbar curve, shoulder girdle retraction is used for high thoracolumbar curves with apex at TH 12 while lumbar curve horizontal side shift is used for low thoracolumbar curve with apex L1. In the case of double-curves both the shoulder retraction and lumbar curve horizontal side shift are combined to attain more symmetrical vertebral column alignment.
  - Training to combine movements in the frontal and sagittal planes to attain cross-sectional derotation.
- Learning trunk muscular stabilisation strategies for self-corrective postures: Patients are instructed on how to recruit deep abdominal and paravertebral muscles through an abdominal hollowing technique to stabilize the trunk. In the case of thoracic curves, simultaneous maintenance of scapular girdle muscle tension and ventralisation of the rib hump is used to reduce the convexity of thoracic curves and facilitate symmetrical thoracic breathing patterns during both inhalation and exhalation. Endurance of isometric contractions with loads that are one-third to two-thirds of maximal loads are trained in sitting and standing positions as well as during walking and activities of daily living.
- Learning over-corrective side shift postural strategies to the opposite side of the primary curve in relaxed sitting and standing positions.
- Patients are instructed to recognize and avoid scoliotic postures by implementing active self-correction and even relaxed over-correction side shift postural strategies as much as possible into daily activities. Patients are asked to monitor their daily compliance to scoliosis specific exercise in a diary.

Session 2

- Reiteration of skills learnt in session 1 and observation or skill progression.
- Training trunk muscular stabilisation and endurance in corrective postures during lower limb closed kinetic chain functional movements such as squats, forward lunges, sideways lunges and single leg standing. The difficulty of each exercise can be increased with growing balance demand in ergonomic lifting, walking, hopping and running situations. The patients are asked initially to perform these specific exercises with 10 repetitions x 3 sets included in their prescription of 60 minutes self-mediated exercise daily. When patients have mastered these exercises, they can instead focus on transferring these skills to similar activities of daily living or individual sporting and recreational activities of interest.

Session 3

- Reiteration of skills learnt in session 2 and observation of skill progression.
- Training trunk muscular stabilisation and endurance in corrective postures during upper and lower limb closed kinetic chain functional movements. The difficulty of each exercise can be increased with growing neuromotor demand such during oculo-manual tasks and sport specific tasks. The patients are asked initially to perform these specific exercises with 10 repetitions x 3 sets included in their prescription of 60 minutes self-mediated exercise daily. When patients have mastered these exercises, they can instead focus on transferring these skills to similar activities of daily living or individual sporting and recreational activities of interest.
- Training of sustained over-corrective side shift mobilisations towards thoracic concavity integrated with the maintenance of sagittal plane curvature and when needed pelvic shift recompensation of the lumbar curve. Balance reactions due to asymmetrical postures and the use of training aids such as a pilates ball, poles or wall bars are used to provide leverage of postural mobilisations and facilitation of muscular stabilization of over-corrective postures. Simultaneous maintenance of scapular girdle muscle tension and ventralisation of the rib hump is used to reduce the convexity of thoracic curves and facilitate symmetrical thoracic breathing patterns during both inhalation and exhalation. Over-corrective mobilization of spinal curvature is conducted always within normal physiological spinal range of movement. Patients are asked to perform over-corrective mobilisations with a dosage of 10 repetitions of 30 second isometric holds daily.

1. *Hyper-corrective night-time brace*

In addition to receiving the same intervention as group 1, a hyper-corrective brace will be worn 8 hours per night. The brace will be specifically designed to provide a 3 dimensional hyper-correction of the patient’s individual scoliosis type. Reinforcement of the assigned intervention will be performed in conjunction with reassessment every 6 months. A training diary will be implemented to follow and motivate the patient's compliance to the intervention protocol. The spine orthotist is available for brace adjustment when needed.
